# Cretan Aging Cohort-Phase III: Methodology and Descriptive Characteristics of a Long-Term Longitudinal Study on Predictors of Cognitive Decline in Non-Demented Elderly from Crete, Greece

**DOI:** 10.3390/healthcare11050703

**Published:** 2023-02-27

**Authors:** Maria Basta, Eleni Skourti, Christina Alexopoulou, Alexandros Zampetakis, Andronikos Ganiaris, Marina Aligizaki, Panagiotis Simos, Alexandros N. Vgontzas

**Affiliations:** 1 Division of Psychiatry and Behavioral Sciences, School of Medicine, University of Crete, 71003 Heraklion, Greece; 2 Sleep Research and Treatment Center, Department of Psychiatry, Penn State University, State College, PA 16802, USA; 3 Department of Intensive Care Unit, University Hospital of Heraklion, 71500 Heraklion, Greece; 4 Computational Biomedicine Lab, Institute of Computer Science, Foundation for Research and Technology-Hellas, 70013 Heraklion, Greece

**Keywords:** elderly, cognitive decline, mild cognitive impairment, RISK factors, mental health indices, longitudinal cohort study

## Abstract

Identifying modifiable factors that may predict long-term cognitive decline in the elderly with adequate daily functionality is critical. Such factors may include poor sleep quality and quantity, sleep-related breathing disorders, inflammatory cytokines and stress hormones, as well as mental health problems. This work reports the methodology and descriptive characteristics of a long-term, multidisciplinary study on modifiable risk factors for cognitive status progression, focusing on the 7-year follow-up. Participants were recruited from a large community-dwelling cohort residing in Crete, Greece (CAC; Cretan Aging Cohort). Baseline assessments were conducted in 2013–2014 (Phase I and II, circa 6-month time interval) and follow-up in 2020–2022 (Phase III). In total, 151 individuals completed the Phase III evaluation. Of those, 71 were cognitively non-impaired (CNI group) in Phase II and 80 had been diagnosed with mild cognitive impairment (MCI). In addition to sociodemographic, lifestyle, medical, neuropsychological, and neuropsychiatric data, objective sleep was assessed based on actigraphy (Phase II and III) and home polysomnography (Phase III), while inflammation markers and stress hormones were measured in both phases. Despite the homogeneity of the sample in most sociodemographic indices, MCI persons were significantly older (mean age = 75.03 years, SD = 6.34) and genetically predisposed for cognitive deterioration (APOE ε4 allele carriership). Also, at follow-up, we detected a significant increase in self-reported anxiety symptoms along with a substantial rise in psychotropic medication use and incidence of major medical morbidities. The longitudinal design of the CAC study may provide significant data on possible modifiable factors in the course of cognitive progression in the community-dwelling elderly.

## 1. Introduction

As life expectancy increases, cognitive impairment becomes an inextricable facet of aging. Worldwide, it is estimated that over 55 million people live with dementia, a number that is about to rise to 139 million people by 2050, while a substantial percentage of dementia patients has yet to receive a formal diagnosis [1]. In contrast, normal cognitive aging comprises predictable age-related cognitive changes, as indicated by age and education-adjusted domain-specific scores that fall within 1.5 standard deviations from the population mean [2]. Persons who display domain-specific (i.e., not global) cognitive impairment, which is not considered serious mental disorder and does not interfere with daily functioning, are likely to be diagnosed with mild cognitive impairment (MCI) [3]. Individuals with MCI are considered at high risk of progression to dementia [4], with conversion rates ranging from 6 to 44.8%, according to a recent meta-analysis [5]. MCI incidence rates increase from 22.5% for ages 75–79 to 60.1% for individuals beyond 85 years old [6]. In Greece specifically, MCI prevalence ranges from 13.11% [7] to 32.4% (Cretan Aging Cohort) [8].

As a prodromal stage of dementia pathology, MCI constitutes a critical “window” for early intervention, and consequently, several studies have focused on identifying modifiable risk factors for cognitive deterioration. Sleep disturbances are a frequent, yet potentially modifiable, comorbid condition in the elderly, which appears to contribute significantly to cognitive impairment and disease prognosis [9]. According to a recent meta-analysis, sleep quality, measured by dysregulation in sleep architecture, was found to differentiate cognitively intact and MCI persons, with the latter group exhibiting increased sleep latency and less Cyclic Alternating Pattern expression compared to healthy individuals [10]. Findings regarding the association between sleep duration and cognitive impairment are rather controversial [11], with some studies indicating greater risk for cognitive decline among short (<6 h) and long sleepers (>8 h), or both [12,13], whilst other studies fail to report such an association. Cross-sectional analyses from the Cretan Aging Cohort (CAC) revealed significant associations between objective long sleep duration and executive deficits among persons diagnosed with MCI and cognitively non-impaired individuals [11], whereas long sleep duration in MCI and Alzheimer’s Disease (AD) patients may be driven by the presence of APOE (Apolipoprotein E) ε4 allele [14].

Other biomarkers (including genetic factors, pro-inflammatory cytokines and stress hormones) contribute to disease progression and differentiate between clinical categories (MCI, dementia). The APOE ε4 allele is an established risk factor for dementia, incident MCI, and rate of conversion from MCI to dementia [15]. Dysregulation of inflammatory response (a condition also known as “inflamm-aging”) seems to play a critical role in the pathogenesis of neurodegenerative diseases, although the underlying mechanisms are not clearly understood [16]. Elevated cerebrospinal fluid and plasma levels of Tumor Necrosis Factor-alpha (TNFa) and Interleukin-6 (IL-6) in AD patients [17] predict further cognitive decline [18] and have been linked to worse cognitive performance in both MCI and AD patients [19]. Impaired regulation of pro-inflammatory cytokine secretion has been found in sleep-related disorders and acute sleep deprivation [20,21]. Moreover, increased IL-6 plasma levels predict poor sleep quality [22] and relate to excessive daytime sleepiness in the cognitively intact elderly [23]. Elevated cerebrospinal fluid and plasma cortisol levels have been detected in both MCI and dementia patients, whereas increased cortisol may exert deleterious effects on memory recall via biphasic activation of specific receptors in the hippocampus, leading to downregulation of Long-Term Potentiation [24]. Additionally, overexpression of cortisol receptors in prefrontal areas may be associated with executive deficits emerging from irregular activity patterns in the prefrontal cortex [25]. The two processes may be interrelated, as impaired executive function mediates the relationship between basal cortisol levels and impaired memory recall [26].

Neuropsychiatric symptoms and mental morbidities are particularly common among elderly with and without neurocognitive disorders or MCI [27,28]. Depression prevalence among MCI patients may be as high as 32% [29] and is considered a risk factor for dementia progression [30] and accelerated rate of cognitive deterioration (possibly moderated by APOE ε4 carriership) [31]. Patients with persistent depressive symptomatology are more likely to present hippocampal atrophy [32], whereas depression diagnosis is often accompanied by pronounced amyloid abnormalities [33]. Anxiety is another frequent comorbid condition (although not as extensively studied as depression), with prevalence rates reaching 21% among MCI patients [34]. Significant anxiety symptoms can compromise daily functioning in MCI patients and increase the risk for dementia progression [35]. A trend towards reduced cognitive performance is present in patients with concurrent anxiety and depressive manifestations, although the contribution of anxiety symptoms on the observed cognitive deficits remains unclear [35]. Anxiety symptoms are also linked to elevated pro-inflammatory cytokines and hypercortisolemia, a condition that leads to dementia-associated brain atrophy due to long-term glucocorticoid exposure [36]. Last but not least, sleep dysregulation is a core depression symptom, and sleep-associated disturbances (insomnia symptoms, poor sleep quality) are overexpressed among MCI patients [37].

The CAC was established in 2013 to investigate sociodemographic, medical, lifestyle, inflammation and neuroendocrine, sleep-related, genetic, cognitive and neuropsychiatric characteristics of the elderly residing in mostly rural areas of the Heraklion prefecture in the island of Crete, Greece. The present report describes the protocol of a 7-year follow-up study on a subset of CAC participants, aimed to identify potentially modifiable predictors of cognitive deterioration among persons who were either cognitively non-impaired or were diagnosed with MCI. Similar large-scale prospective studies are being conducted in Greece and focus on sociodemographic information, medical and mental health indices, lifestyle factors and biomarkers (SHARE; Survey of Health, Ageing and Retirement in Europe [38,39]), as well as nutrition and neuropsychological markers of cognitive progression (HELIAD study; Hellenic Longitudinal Investigation of Aging & Diet [40]). However, to our knowledge, up to now, this is the first longitudinal cohort study conducted in Greece and among few worldwide with a relatively large, well-defined sample—including MCI patients—with a special focus on objective sleep, inflammation, stress and neuropsychiatric symptoms as possible modifiable factors for dementia.

## 2. Materials and Methods

### 2.1. Study Design

#### 2.1.1. Phase I–Phase II

During Phase I, 3140 community-dwelling participants (mean age 73.7 ± 7.8 years) [8] from rural areas of Heraklion, Crete (Cretan Aging Cohort) were examined. Eligible participants were those aged ≥60 years old who visited Primary Health Care Centers (staffed by physicians participating in the Primary Health Care research network of the CAC study) in both rural and urban areas of Heraklion and consented to participate in the study. Patients with acute symptomatology (terminal illnesses, severe movement impairment) were excluded from the study. Data from the 2011 national census were utilized in order to compare CAC participants to the whole Greek and Cretan population of similar age (for a more thorough analysis, see [8]). Demographic information and medical data were collected, and all participants were administered the Mini Mental State Examination (MMSE) test. Participants who had scored <24 points on MMSE (n = 636) were invited to a comprehensive neuropsychological and neuropsychiatric examination (Phase II), and a total of 344 consenting persons (comparable in terms of demographic and anthropometric measurements to the 636 participants) completed the evaluation. A control group (n = 181) of persons scoring ≥24 points on MMSE during Phase I was also formed using a proportional stratification process to match the low MMSE group on gender and place of residence. Of those, 161 persons consented and took part in Phase II examination [11]. During Phase II (2013–2014), all participants underwent full neuropsychological/neuropsychiatric/neurological evaluation, 3-day, 24-h actigraphy recording, and blood sampling (to measure baseline morning cortisol, pro-inflammatory cytokines and genetic biomarkers); medical history, sleep complaints and general functionality information were also recorded. Consensus clinical diagnoses for dementia and MCI were based upon the Diagnostic & Statistical Manual of Mental Disorders (DSM, 4th Edition) and the International Working Group (IWG) criteria, accordingly [8]. In total, 146 persons were found cognitively intact, whilst 231 participants were diagnosed with MCI of any type [8].

#### 2.1.2. Phase III

**Figure 1:**
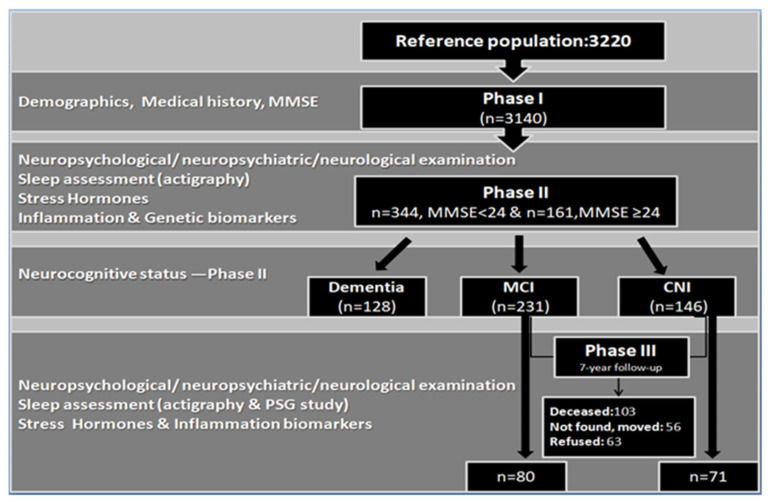
Flow diagram of Phases I, II & III of the Cretan Aging Cohort study. Phases I & II were conducted within approximately six months in 2013, whereas Phase III assessments were conducted between 2020–2022. Participant diagnostic status during Phase II is also shown. Abbreviations; MMSE: Mini Mental State Examination, MCI: mild cognitive impairment, CNI: cognitively non-impaired, PSG: polysomnography.

The participant pool for the 7-year follow up study (Phase III) comprised all CNI persons (n = 146) and individuals who met the formal criteria for MCI (n = 231) during Phase II. Patients diagnosed with dementia were excluded from Phase III testing, which took place between October 2020 and August 2022 (see Figure 1). In total, 103 participants (27.3%) had passed away in the intervening years, 56 persons (14.9%) could not be located, and 63 persons (16.7%) refused to participate, raising the total attrition rate (inability to participate for any reason) to 58.9%. In total, 149 MCI and 73 CNI individuals could not be retested. From the 274 survivors, 155 individuals completed the evaluation, although data from four participants were not included in the analyses due to severe medical comorbidities or sensory loss. Thus, the final response rate reached 55.1%. All participants were contacted by telephone and came from 11 different districts in the prefecture of Heraklion. Testing procedures were similar to those followed in Phase IΙ, permitting direct quantitative comparisons between the two time points on the majority of measures. Examination was conducted at participants’ homes and included medical history and physical examination, neuropsychological testing, a night of polysomnography recording and a 7-day, 24-h actigraphy, as well as a morning blood draw to assess stress and inflammatory biomarkers. The study was approved by the Ethics Committee of the University of Crete (number of approval: 61/9-3-2020). A detailed description of the study protocol is provided below. Figure 1 presents a flow chart of the entire study.

### 2.2. Measurements

#### 2.2.1. Sleep Measurements

(i) Polysomnography (PSG)

We collected data from 144 participants. Each participant underwent one night home sleep study ad libitum using a portable Type II7 16 channel polysomnography device (Alice, PDx, Philips, Respironics, Murrysville, PA, USA). The sleep study registered the following parameters: oral-nasal airflow via pressure cannula and thermistor, respiratory effort via the abdominal and chest belts, arterial oxygen saturation level via the pulse oximeter (oxygen saturation and pulse rate), body position detection (supine or non-supine), cardiac electrical activity, C3M2 and C4M1 electroencephalogram, electrooculogram and chin and leg electromyogram. Scoring was performed manually from a sleep expert physician according to the American Association Sleep Medicine scoring manual version 2.6.2020. Apnea/Hypopnea episodes followed the standard procedures (AASM, 2007) and Obstructive Sleep Apnea was defined as an Apnea/Hypopnea Index ≥ 15. Additional sleep variables, such as Sleep Latency, Total Sleep Time, Total Time in Bed, Sleep Efficiency and Wake Time after Sleep Onset were also scored according to the standard AASM 2007 criteria.

(ii) Actigraphy

The majority of participants (n = 110) completed a 7-day, 24-h wrist actigraphy recording (Actigraph, GT3XP model, Pensacola, FL, USA) as a complementary means to estimate sleep duration and quality, using the same procedures followed in Phase II [11]. Sleep–wake cycle estimation was based on epochs of movement (peaks of activity) or movement absence (relatively quiet periods of activity) using the ActLife 6 software (ActLife v6.9.5 LLC, Pensacola, FL, USA) and complemented by sleep diaries. Data were collected and averaged for the 7-day and 3-day period separately, and specific variables of interest were calculated: night and 24-h total sleep time, night and 24-h total time in bed, sleep latency and efficiency, wake time after sleep onset, and number and mean duration of night awakenings. For 104 participants, actigraphy took place simultaneously or within 24 h from PSG recording. Six participants underwent actigraphy recording within 1–4 months from PSG recording due to technical issues.

#### 2.2.2. Inflammatory Biomarkers

Single morning blood samples were collected (between 10:00 am and 12:00 pm) to assess inflammatory markers (IL-6, TNFa and C-Reactive Protein, n = 119) and plasma cortisol levels (available for116 participants). Blood samples were transferred to EDTA-containing tubes, refrigerated, centrifuged for plasma isolation and kept in deep freeze (−80 °C). Plasma TNFa and IL-6 were measured using the ELISA technique (Human TNF-alpha Quantikine HS ELISA and Human IL-6 Quantikine HS ELISA kits, R&D Systems Europe, Abington, UK). Plasma cortisol levels were measured using the ELISA technique (Cusabio Technology LLC, Texas, USA). The same procedure was followed at Phase II, rendering results comparable between the two phases [41].

#### 2.2.3. Diagnosis of Neurocognitive Impairment

(i) Neuropsychological assessment

All participants underwent a thorough neuropsychological examination (mean duration ≤ 2.5 h). Domains evaluated included memory (episodic and verbal memory: Greek Memory Scale and Rey Auditory Verbal Learning Test, respectively; spatial memory: Taylor Complex Figure and working memory: Digits Reverse), language (naming ability: Boston Naming Test-short version and verbal fluency: the Semantic Verbal Fluency test) and attention/executive function (processing speed: Symbol Digits Modality test and visuomotor speed, task shifting and selective attention: Trails A & B). Raw scores were transformed into age and education-standardized values (based on normative values), and average z-scores on each cognitive domain were computed. Impaired performance on a given domain was considered if the average z-score was at least 1.5 SD below normative values. For the diagnosis of MCI, impaired performance in two or more tests within a given cognitive domain and intact functionality level (based on an Independent Activities of Daily Living (IADL) score > 0.78) were required. In cases of severe cognitive impairment, the MMSE test was administered instead. A Clinical Dementia Rating score was also calculated to aid cognitive status classification, especially in cases of severe cognitive impairment and significant sensory limitation.

(ii) Informant scales

Close relatives or caregivers were asked to complete scales measuring daily functioning (the 13-item Greek Independent Activities of Daily Living scale), current cognitive and neuropsychiatric symptoms (Cambridge Behavioral Inventory) and symptoms indicative of Lewy-body dementia (4-item Mayo Fluctuations Scale). An average IADL score < 0.78 points (range 0 to 1.00) was considered as indicative of significant functional impairment, a core criterion of severe cognitive impairment diagnosis (Dementia of any type). According to the IWG criteria, MCI diagnosis requires intact basic daily activities and relatively preserved instrumental daily functioning. Therefore, an IADL score > 0.78 points serves as a marker of adequate/preserved daily functionality in persons with mild cognitive impairment and CNI individuals.

#### 2.2.4. Semi-Structured Interview

A comprehensive medical history was taken, including the following domains that were initially assessed at baseline:

- Current and past medical conditions, with emphasis on illnesses and operations occurring during the follow-up period, including Traumatic Brain Injury (TBI), stroke and pharmacotherapy (any type of treatment with a special focus on psychotropic substances). We then calculated total number of major medical morbidities (hypertension, diabetes, heart/pulmonary/hematological/liver diseases, gastrointestinal conditions, hyper/hypothyroidism, cancer, arthritis).
- Mental morbidities (i.e., depression and anxiety diagnosis) were assessed according to the DSM-5 criteria, based on a clinical interview, neuropsychological evaluation, and existing diagnosis following the same procedures described previously [28].
- Anthropometric measurements: weight, height, and Body Mass Index were assessed as previously described [8].
- A frailty composite index was calculated based on level of physical activity, self-reported symptoms of exhaustion and decreased appetite, and objectively assessed upper limb weakness (using a dynamometer measurement). Frailty level was then recorded into 3 classes (absence of frailty, pre-frailty, frailty).
- Overall subjective memory difficulties were assessed via a single question (“Do you have any memory problems?”), requiring a yes/no response, whereas domain-specific memory complaints (difficulty recalling recent information, words and names) were assessed using single questions requiring a binary response (WHICAP medical package: Medical Conditions and WHICAP survey).
- Sleep problems: we used a shortened version of the Penn State Sleep Questionnaire comprising 12 items (answered on a 4-point Likert scale ranging from 0 = absence of symptoms to 3 = serious symptomatology) in order to assess presence and severity of self-reported sleep complaints, sleep duration and napping throughout the day (apnea, snoring, excessive movements during sleep, difficulty falling/staying asleep, early awakening, overall quality of sleep and, lastly, average night sleep duration and time required for falling asleep, as well as napping frequency and duration, if applicable) [41].
- Lifestyle habits: we recorded current smoking and drinking habits (number of cigarettes if a current smoker, smoking cessation and year of quitting, as well as frequency of alcohol consumption on a daily basis). We also estimated level of physical activity during the previous week (including frequency of participation in particular activities such as gardening, housework, handiwork, shopping), as well as based on participants’ responses to the question “How many days did you walk for more than 10 min in a row in a brisk manner during the last week?”, as previously described in detail [41].
- Social support and frequency of social contacts: we calculated the total number of social contacts (close relatives and friends) reported by participants during the last month, the availability of emotional and practical support, using two questions adapted from the Social Support Questionnaire–Short Form [42]: “Is there anyone you can really count on when you need help? Is there anyone you can really count on to help you feel more relaxed when you are under pressure/stress?” and the quality of perceived support (“How satisfied are you with the level of support you receive?”), answered on a 5-point Likert scale ranging from 0 (not at all) to 4 (completely satisfied).

#### 2.2.5. Neuropsychiatric Evaluation

Self-reported symptoms of anxiety and depression were assessed using the 7-item Hamilton Depression and Anxiety Scale-Anxiety subscale (HADS-A) and the 15-item Geriatric Depression Scale (GDS), respectively. Diagnosis of depression and anxiety during Phase III followed the same procedure as in Phase II, according to the DSM-5 criteria established through a clinical interview conducted by a specially trained physician and psychologist, scores on the aforementioned scales (using 7 and 4 points as cutoffs, respectively) and prescription of psychotropic medication(antidepressants/anxiolytics or antipsychotics) [28]. Furthermore, in Phase III, we recorded retrospectively major stressful events that occurred within the 7-year interval and calculated a new binary variable to indicate the presence of at least one major stressor in the period preceding the examination process. Major stressors included significant medical conditions (severe eyesight/hearing loss, cancer), death or illness of close relatives and finally, survival from natural disasters (there was consecutive severe and frequent earthquake activity in Crete in the time preceding Phase III assessment).

Following the same procedures as in Phase II, all relevant information (cognitive performance by domain, IADL score, neuropsychiatric symptoms) was evaluated by a certified psychiatrist (M.B), neurologist (C.C.) and neuropsychologist (P.S) to reach a consensus diagnosis according to theDSM-4 and DSM-5 criteria (for Phase II and III accordingly) for the diagnosis of Major Neurocognitive Disorder and the IWG criteria for the MCI diagnosis [43]. Dementia differential diagnosis was made on the basis of the following criteria: for the diagnosis of probable AD, vascular Dementia, Lewy Body Dementia, behavioral variant FTD and other types of Frontotemporal Dementia, the NINCDS-ADRDA, the NINDS-AIREN, the DLB Consortium, the International Consortium on behavioral variant Frontotemporal Dementia and the Neary criteria were utilized, accordingly [44,45,46,47,48]. Diagnosis of mixed dementia was made in cases of co-occurrence of signs suggestive of both probable AD and vascular dementia [49].

### 2.3. Statistical Analysis

SPSS 28.0 (IBM; 2022) was used for statistical analyses. In view of significant deviation from normality for a number of variables (as indicated by *p* < 0.05 on the Kolmogorov–Smirnov test), non-parametric tests (Wilcoxon signed-rank test and Mann–Whitney U test) were used to assess change over time and group differences at each Phase, respectively. The Chi square test was used to assess differences in proportions. The final sample size was sufficient to ensure 85% power for detecting small-to-medium effect size group differences at *p* < 0.05 and also sufficient to ensure 95% power for detecting small effect sizes of change over time at *p* < 0.05.

## 3. Results

Seventy-one CNI and 80 participants previously diagnosed with MCI in Phase II were re-evaluated in Phase III at an average interval of 7.12 years (SD = 0.92). Compared to the total participant pool (all persons in the CNI and MCI groups in Phase II, n = 377), those who were followed up were younger (72.8 vs. 77.2 years, *p* < 0.001), more likely to be women (77.5% vs. 63.3%, *p* = 0.004) and less likely to live alone (*p* = 0.03). There was a non significant tendency for followed-up persons to have achieved more years of education (*p* = 0.059). The total group and followed-up subgroup were comparable in terms of geographic origin (*p* = 0.4), major medical morbidities (*p* = 0.9) and previous occupation (*p* = 0.1). As evident in Table 1, the majority of participants in the current cohort were rural residents (84.1%), previously occupied in domestic/agricultural work (63.6%) and having attained 6 or fewer years of formal education (92.1%).

In Phase II, the two diagnostic groups (i.e., CNI, MCI) were comparable in Body Mass Index, gender ratio, lifestyle habits, previous occupation, frequency of persons living alone, overall health (as indexed by the number of current major medical morbidities), and family history of dementia (see Table 1), with the exception of age (CNI < MCI, *p* < 0.001) and frequency of APOE ε4 carriers (CNI < MCI, *p* = 0.04). Moreover, the two diagnostic groups did not differ in psychiatric manifestations (severity of self-reported anxiety and depression symptoms, depression and anxiety diagnosis) or frequency of psychotropic medication use (see Table 2). In Phase III, the two groups were comparablein all variables. Occurrence of major stressors during the follow-up period was also very similar between the two groups, as was the frequency of persistent depression diagnosis (21.1 vs. 17.5% for CNI and MCI, respectively, *p* = 0.6).

Over the follow-up period, participants in both groups reported increased anxiety symptoms (*p* < 0.001), although the frequency of anxiety diagnosis did not vary significantly (*p* = 0.6 and *p* = 0.2 within the CNI and MCI groups, respectively). This trend was paralleled by a concurrent increase in the use of at least one psychotropic medication, which reached significance in both groups (*p* < 0.001 and *p* = 0.005 in the CNI and MCI group, respectively). Whereas self-reported depression symptoms did not vary significantly across the two time points between CNI and MCI groups, the frequency of depression diagnosis changed significantly over time within diagnostic groups (increasing trend, statistically significant among CNI persons, *p* < 0.001). Alcohol use was reduced (*p* = 0.028 and *p* = 0.023 in CNI and MCI group, respectively). Finally, there was an increase in those living alone within the CNI group (*p* = 0.003) and in the average number of major medical morbidities in both groups (*p* = 0.001 and *p* < 0.001 in CNI and MCI groups, respectively), possibly as a result of aging.

## 4. Discussion

In this paper, we outline the study protocol and the sociodemographic, medical and mental health characteristics of the sample of a 7-year longitudinal study on aging, aiming to identify predictors of cognitive decline in community-dwelling elderly participants. The sample derived from the CAC included persons averaging 72.9 (range: 60–89) years old at baseline who either met criteria for MCI or were cognitively intact upon initial examination. Considering the age range of participants, we achieved satisfactory response rate (55.1%) in this well-characterized, culturally homogeneous, mainly rural (84.1%), low-literacy sample (92.1% had completed ≤6 years of formal education). This longitudinal study is rather unique as it involves multimodal measurements of a wide range of factors, which could act as either direct predictors of cognitive decline or as moderators of the impact of other variables on long-term cognitive status progression in this well-defined community-dwelling elderly sample.

Few studies have investigated the interplay between sleep abnormalities, mental and physical comorbid disorders, inflammatory biomarkers, stress-related hormones, behavioral/psychological symptoms and domain-specific cognitive performance among persons diagnosed with different levels of cognitive and functional impairment longitudinally. Until recently, the majority of actigraphy and polysomnography studies recruited small groups of cognitively intact and MCI participants [10]. To our knowledge, this is the first longitudinal study conducted in Greece and among few studies worldwide that uses several qualitative and quantitative measures, providing an objective, integrative assessment of sleep patterns, sleep-related disorders (Obstructive Sleep Apnea) and sleep macrostructure, as well as their interplay with cognitive performance and possible confounding factors (inflammatory and genetic biomarkers, mental and physical comorbidities, sociodemographic and lifestyle conditions)in a relatively large sample.

The two diagnostic groups (CNI and MCI) were relatively similar in sociodemographic, medical and emotional conditions at baseline, including family history of dementia, except that MCI persons were older and more likely to be APOE ε4 allele carriers. At follow-up, we noted a significant increase in the number of major medical morbidities, which is expected with advancing age. In terms of mental health, both groups reported increased severity of anxiety symptoms and use of psychotropic medications (anti-depressants and anxiolytics), possibly as a consequence of aging as well as the long-term and ongoing effects of two consecutive crises, namely the Greek financial crisis of 2009–2019, which resulted in further income reductions and increased unemployment, and the global pandemic crisis, which caused insecurity and exacerbated feelings of distress among Greeks [50,51]. Furthermore, depression diagnosis (based on the clinical interview and antidepressant prescription criteria) was notably increased at re-evaluation, especially among cognitively non-impaired persons. It should be stressed, though, that subjectively assessed depressive symptomatology remained relatively stable between the two measurement points (as opposed to increased frequency of depression diagnosis), assumingly due to increased anti-depressant use, which led to symptom alleviation at follow-up. Depression and anxiety are frequent comorbid conditions among the elderly, and their co-occurrence increases the chance of somatic symptoms and cognitive deterioration [52]. Development of depression and anxiety symptomatology is closely related to multimorbidity [35], presence of chronic illnesses, and stressful life events [52]. The number of medical morbidities increased in Phase III, and at the same time, one out of three participants reported at least one type of major stressful event. Major stressors that trigger feelings of threat or undermine functional independence (as in the case of severe sensory loss) predict both depressive and anxiety symptoms [53].

Given the demographic characteristics of the current population (low educational level and rural residence), lack of familiarity with the utilized techniques (actigraphy and polysomnography), the time-consuming nature of the study procedures and the lack of personal incentives (i.e., remuneration), the response rate can be considered satisfactory. Our project was delayed for 7 months due to COVID-19 pandemic restrictions, whereas excessive worrying about COVID infection during examination and/or inconsistent information about the effectiveness of protective measures against coronavirus expansion may have negatively affected the response rate. However, despite the adverse conditions and the insurmountable challenges posited by the pandemic, the Phase III response rate (51.1%) was among the highest compared to similar studies conducted in Greece [40] and Southern Europe [54].

Lastly, some limitations of the current protocol should be discussed. Despite the fact that all testing procedures took place in participants’ homes to reduce the inconvenience of a hospital visit and to increase ecological validity, we could not control for the presence of environmental distractors during neuropsychological testing (although we opted for a distraction-free environment), fatigue or reduced compliance with the instructions pertaining to the polysomnographic process. In addition, although home PSG is a well-validated process for sleep assessment, it is associated with artifacts and data loss due to lack of continuous monitoring by overnight technical staff.

## 5. Conclusions

The current study aimed to identify modifiable risk factors for cognitive deterioration by embracing a comprehensive, multidisciplinary approach, utilizing user-friendly techniques. This is important given the high progression rates from MCI to dementia, the urgent need for timely interventions, as well as the complex interplay between risk factors for cognitive decline. Strengths of this study include the longitudinal design, the relatively large number of MCI patients recruited, the particular socio-economic and cultural characteristics of the current sample, the long follow-up interval and methodological advantages (presence of a control group), which we expect to result in scientifically valid and clinically useful findings in terms of modifiable factors predisposing to cognitive progression among the elderly.

## Figures and Tables

**Table 1:** Sociodemographic and medical characteristics assessed in Phase II and III for the cognitively non-impaired (CNI) and MCI participants (according to Phase II diagnosis).

KERRYPNX	CNI (n = 71)		MCI (n = 80)		MCI vs. CNI	MCI vs. CNI
	Phase II	Phase III	Phase II	Phase III	(Phase II)	(Phase III)
Age (years)	70.48 (6.31)	78.32 (6.16) *	75.03 (6.34)	83.30 (6.27) ^†^	<0.001	<0.001 ^1^
Gender (Female, (%))	55 (77.5)		62 (77.5)		0.9 ^2^	
RuralResidence (%)	59 (83.1)		68 (85.0)		0.7	
Body Mass Index	31.22 (4.22)	31.10 (5.89)	30.12 (4.55)	30.05 (5.95)	0.07	0.3
Living alone (%)	17 (23.2)	23 (32.4) *	16 (20.0)	18 (22.5)	0.6	0.2
No of Illnesses	2.55 (1.62)	3.28 (1.62) *	2.49 (1.37)	3.18 (1.50) ^†^	0.8	0.5
Education (years)	5.49 (3.23)		4.70 (2.55)		0.06	
Previous occupation (%)					0.6	
Housekeeping	13 (18.3)		22 (27.5)			
Farmer	28 (39.4)		33 (41.2)			
Worker	7 (9.9)		9 (11.2)			
Technician	1 (1.4)		1 (1.3)			
Employee	11 (15.5)		6 (7.5)			
Self-employed	9 (12.7)		8 (10.0)			
Teacher	2 (2.8)		1 (1.3)			
Dementia Family history (%)	20 (28.2)		24 (30.0)		0.8	
APOE ε4 allele (%)	6 (8.5)		19 (24.4)		0.04	
Smoking (%)	7 (9.9)	6 (8.6) *	3 (3.8)	2 (2.5) ^†^	0.1	0.1
Alcohol use (%)	21 (29.6)	13 (18.8) *	35 (43.6)	16 (20.0) ^†^	0.08	0.8

^1^ Mann–Whitney U test, ^2^ Chi square test of independence. Notes: Significant differences (*p* < 0.05) between the two time points within the same diagnostic group are indicated by * (CNI group) or ^†^ (MCI group). Abbreviations; CNI: cognitively non-impaired, MCI: mild cognitive impairment, APOE: Apolipoprotein E. Unless otherwise specified, values are mean (SD).

**Table 2:** Mental health characteristics assessed in Phase II and III for cognitively non-impaired (CNI) and MCI participants (according to Phase II diagnosis).

	CNI (n = 71)		MCI (n = 80)		MCI vs. CNI	MCI vs. CNI
	PhaseII	Phase III	PhaseII	Phase III	(Phase II)	(Phase III)
HADS-A subscale score	3.57 (3.71)	5.83 (4.28) *	2.81 (3.09)	4.79 (3.63) ^†^	0.5	0.2 ^1^
GDS score	3.84 (3.62)	3.59 (2.88)	3.93 (3.07)	3.58 (3.05)	0.8	0.9
Depression Diagnosis (%)	20 (28.2)	26 (36.6) *	27 (33.8)	30 (37.5)	0.5	0.9 ^2^
Anxiety Diagnosis (%)	19 (26.8)	26 (36.6)	26 (32.5)	20 (25.0)	0.4	0.1
Psychotropic medication use (%) ^3^	26 (36.6)	32 (45.1) *	21 (26.3)	36 (45.6) ^†^	0.2	0.9
Persistent Depression (%) ^4^	15 (21.1)		14 (17.5)		0.6	
Major stressful events (%) ^4^	21 (29.6)		30 (37.5)		0.3	

^1^ Mann-Whitney U test, ^2^ Chi square test of independence, ^3^ Antidepressants, anxiolytics, antipsychotics, ^4^ 7-year interval. Notes: Significant differences (*p* < 0.05) between the two time points within the same diagnostic group are indicated by * (CNI group) or ^†^ (MCI group). Abbreviations; CNI: cognitively non- impaired, MCI: mild cognitive impairment, HADS-A: Hamilton Anxiety & Depression Scale-Anxiety subscale, GDS: Geriatric Depression Scale. Unless otherwise specified, values are mean (SD).

## Data Availability

Data available on request due to restrictions (privacy). The data presented in this study are available on request from the corresponding author. The data are not publicly available due to privacy restrictions.
